# Correction: An amplification-free, 16S rRNA test for *Neisseria gonorrhoeae* in urine

**DOI:** 10.1039/d3sd90005c

**Published:** 2023-01-30

**Authors:** Zhenrong Zheng, Yan Cao, Sukantha Chandrasekaran, Jacob J. Schmidt, Omai B. Garner, Harold G. Monbouquette

**Affiliations:** a Chemical and Biomolecular Engineering, University of California, Los Angeles Los Angeles CA 90095 USA hmonbouq@ucla.edu; b Pathology and Laboratory Medicine, University of California, Los Angeles Los Angeles CA 90095 USA; c Bioengineering, University of California, Los Angeles Los Angeles CA 90095 USA

## Abstract

Correction for ‘An amplification-free, 16S rRNA test for *Neisseria gonorrhoeae* in urine’ by Zhenrong Zheng *et al.*, *Sens. Diagn.*, 2023, **2**, 163–167, https://doi.org/10.1039/d2sd00128d.

The authors regret to report that the PNA probe used to detect *N. gonorrhoeae* is selective for 16S rRNA gene template strand DNA, not 16S rRNA as reported. Corrections should be made throughout the manuscript, including the Fig. 2 caption. However, the detection data shown in Fig. 2 and in Table 1 are not affected. It also is important to note that DNase was not used when extracting nucleic acid thereby permitting detection of the 16S rRNA gene DNA.

As a result of this change, the following corrections should be made throughout the manuscript:

In the first sentence of the **Abstract**, “16S rRNA” should be changed to “16S rRNA gene”. Later in the **Abstract**, “*N. gonorrhoeae* 16S rRNA” should be changed to “*N. gonorrhoeae* 16S rRNA gene DNA”.

In the last sentence of the paragraph on **Detector assembly** under **Methods**, “target *N. gonorrhoeae* 16S rRNA” should read, “target *N. gonorrhoeae* 16S rRNA gene DNA”.

In the first sentence of the paragraph on **Coupling PNA probe to microspheres** under **Methods**, “detecting *N. gonorrhoeae* 16S rRNA”, should read, “detecting *N. gonorrhoeae* 16S rRNA gene template strand DNA”.

The paragraph on **RNA extraction** under **Methods** should be re-titled, “**NA extraction**”. Also where “total RNA” appears it should be changed to “NA”. The phrase, “without DNase treatment” should be inserted after “(Zymo Research, Irvine, CA)”. Finally, in the last line of this paragraph “RNase-free” should be changed to “DNase/RNase-free”.

The paragraph on **Hybridization of RNA to PNA-bead conjugates** under **Methods** should be re-titled, “**Hybridization of 16S rRNA gene DNA to PNA-bead conjugates**”. In the third sentence of this paragraph, “Subsequently, extracted RNA” should be changed to “Subsequently, extracted NA”. Finally, in this paragraph, “16S rRNA” should be changed to “16S rRNA gene DNA”.

In the paragraph on **Sample detection** under **Methods**, “16S rRNA” should be changed to “16S rRNA gene DNA” in two locations.

In the Fig. 2 caption, “16S rRNA” should be changed to “16S rRNA gene DNA” in two locations. Also, “response to RNA extracted” should be changed to “response to NA extracted”. Finally, “RNA extracted from” should be changed to “NA extracted from”.

In the second paragraph of **Results and discussion**, “RNA instability” should be changed to “DNA instability”, while in the third paragraph, “RNA extraction” should be changed to “NA extraction”.

In the first line of the **Conclusions**, “rRNA test” should be changed to “rRNA gene test”.

The Royal Society of Chemistry apologises for these errors and any consequent inconvenience to authors and readers.

## Supplementary Material

